# The risk of dementia in multiple sclerosis and neuromyelitis optica spectrum disorder

**DOI:** 10.3389/fnins.2023.1214652

**Published:** 2023-06-15

**Authors:** Eun Bin Cho, Se Young Jung, Jin-Hyung Jung, Yohwan Yeo, Hee Jin Kim, Kyungdo Han, Dong Wook Shin, Ju-Hong Min

**Affiliations:** ^1^Department of Neurology, College of Medicine, Gyeongsang Institute of Health Science, Gyeongsang National University, Jinju, Republic of Korea; ^2^Department of Neurology, Gyeongsang National University Changwon Hospital, Changwon, Republic of Korea; ^3^Department of Family Medicine, Seoul National University Bundang Hospital, Seongnam, Republic of Korea; ^4^Department of Digital Healthcare, Seoul National University Bundang Hospital, Seongnam, Republic of Korea; ^5^Samsung Biomedical Research Institute, Sungkyunkwan University School of Medicine, Suwon, Republic of Korea; ^6^Department of Family Medicine, College of Medicine, Hallym University Dongtan Sacred Heart Hospital, Hwaseong, Republic of Korea; ^7^Department of Neurology, Samsung Medical Center, Sungkyunkwan University School of Medicine, Seoul, Republic of Korea; ^8^Neuroscience Center, Samsung Medical Center, Seoul, Republic of Korea; ^9^Department of Health Sciences and Technology, Samsung Advanced Institute for Health Sciences and Technology (SAIHST), Sungkyunkwan University, Seoul, Republic of Korea; ^10^Department of Statistics and Actuarial Science, Soongsil University, Seoul, Republic of Korea; ^11^Department of Family Medicine and Supportive Care Center, Samsung Medical Center, Sungkyunkwan University School of Medicine, Seoul, Republic of Korea; ^12^Department of Clinical Research Design and Evaluation, Sungkyunkwan University, Seoul, Republic of Korea; ^13^Department of Digital Health, Samsung Advanced Institute of Health Science and Technology (SAIHST), Sungkyunkwan University, Seoul, Republic of Korea; ^14^Center for Wireless and Population Health Systems, University of California San Diego, La Jolla, CA, United States

**Keywords:** multiple sclerosis, neuromyelitis optica spectrum disorders, dementia, risk, population

## Abstract

**Introduction:**

Cognitive impairment is a common feature of multiple sclerosis (MS) and neuromyelitis optica spectrum disorder (NMOSD). However, there is a lack of population-based study of dementia risk in these disorders. In the present study, the risk of dementia in MS and NMOSD patients in Republic of Korea was estimated.

**Methods:**

Data analyzed in this study were obtained from the Korean National Health Insurance Service (KNHIS) database between January 2010 and December 2017. The study included 1,347 MS patients and 1,460 NMOSD patients ≥40 years of age who had not been diagnosed with dementia within 1 year prior to the index date. Matched controls were selected based on age, sex, and the presence of hypertension, diabetes mellitus, or dyslipidemia.

**Results:**

In MS and NMOSD patients, the risk of developing any dementia [adjusted hazard ratio (aHR) = 2.34; 95% confidence interval (CI) = 1.84–2.96 and aHR = 2.19; 95% CI = 1.61–3.00, respectively], Alzheimer’s disease [AD; aHR = 2.23; 95% confidence interval (CI) = 1.70–2.91 and aHR = 1.99; 95% CI = 1.38–2.88, respectively], and vascular dementia (aHR = 3.75; 95% CI = 1.91–7.35 and aHR = 3.21; 95% CI = 1.47–7.02, respectively) was higher compared with the matched controls. NMOSD patients had a lower risk of any dementia and AD compared with MS patients after adjusting for age, sex, income, hypertension, diabetes, and dyslipidemia (aHR = 0.67 and 0.62).

**Conclusion:**

The risk of dementia increased in MS and NMOSD patients and dementia risk was higher in MS than in NMOSD.

## Introduction

Multiple sclerosis (MS) and neuromyelitis optica spectrum disorder (NMOSD) are chronic inflammatory demyelinating diseases of the central nervous system (CNS) involving the optic nerve, spinal cord, and brain. Cognitive impairment frequently occurs even in the early stage of disease and exists in association with the disruption of white matter (WM) networks and gray matter atrophy (Bergsland et al., 2012; Liu et al., 2015; Cho et al., 2018; Oertel et al., 2019; Benedict et al., 2020). Worsening of cognitive impairment becomes more prominent with neurodegeneration in the progressive stage of MS (Benedict et al., 2020). In adult patients with MS, the prevalence of cognitive deficit ranges from 30 to 70% depending on study population and definition of cognitive impairment (Benedict et al., 2020). The pooled prevalence of cognitive impairment is 44% in NMOSD, although secondary progression is extremely rare (Moghadasi et al., 2021).

Slowed information processing speed and new learning disabilities are key features of impaired cognition in both MS and NMOSD (Cho et al., 2018; Benedict et al., 2020), which differ from memory impairment in Alzheimer’s disease (AD). However, as patients age, other types of neurodegenerative dementia may overlap with disease-specific cognitive deficits, exacerbating the rate of cognitive decline. Impaired verbal fluency has been observed in elderly patients with MS (average age, 62 years), which is not typical of MS-associated cognitive deficits (Jakimovski et al., 2019). However, knowledge regarding the association between CNS demyelinating diseases and dementia of other pathologies, such as AD or vascular dementia, is limited. Cognitive impairment affects quality of life (Benito-Leon et al., 2002; Lopez-Soley et al., 2022) and superimposed AD may increase socioeconomic burden and mortality (Black et al., 2018) in patients with MS and NMOSD. In addition, the treatment modalities for cognitive decline are different between MS and AD. While cognitive dysfunction in MS can be modulated by disease-modifying drugs and cognitive rehabilitation (Chen M. et al., 2021), there are specific drugs for AD treatment. Therefore, the coexistence of these disorders not only needs to be recognized, but it is important to distinguish them from one another.

In the present study, the risk of dementia in MS and NMOSD patients was compared with matched controls using the Korean National Health Insurance Service (KNHIS) database. In addition, we tried to differentiate the type of dementia developed in these patients.

## Materials and methods

### Data source and study setting

The data analyzed in this study were obtained from the KNHIS database.^1^ KNHIS is the national single insurer and covers approximately 97% of the population in Republic of Korea, except for 3% of Medicaid recipients (Shin et al., 2016). The KNHIS database includes sociodemographic information including age, sex, residential area, and household income level. The qualification of enrolled subjects is managed by linking the database to a death registry of Statistics Korea. In addition, the KNHIS database has information on hospital visits, prescriptions, and diagnoses coded according to the International Classification of Diseases 10th revision (ICD-10), which are identified and collected via claim and reimbursement process.

### Definition of MS and NMOSD and control matching

The MS and NMOSD definitions were adapted from a previous study in which the prevalence of MS and NMOSD in Republic of Korea was investigated (Kim et al., 2020). In that study, patients with MS or NMOSD were defined using both ICD-10 diagnosis codes and inclusion in Rare Intractable Diseases (RID) registration program of Korea, which began in 2009. If patients are diagnosed with diseases applicable to RID, the Korean government reimburses 90% of treatment and work-up expenses for the diseases. Physicians are required to examine patients and certify the diagnosis strictly based on the 2005 McDonald criteria for MS (Polman et al., 2005) or 2006 Wingerchuk criteria (2010–2015) and/or revised 2015 NMOSD diagnostic criteria (after 2016) (Wingerchuk et al., 2006, 2015) for NMOSD to submit an application to the KNHIS for MS or NMOSD. This rigorous registration process ensures the reliability of MS and NMOSD diagnoses. Patients with MS or NMOSD were included in the present study if they met the following criteria: two outpatient or hospitalization claims with the specific ICD-10 code (G36.0 for NMO and G35.0 for MS), and enrollment in the RID program with the diagnosis code for MS or NMOSD.

**FIGURE 1:**
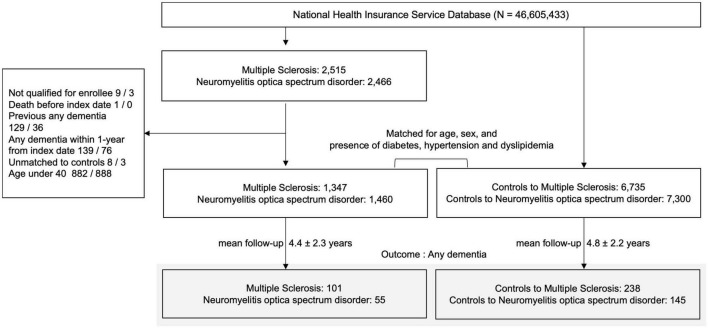
Enrollment flowchart of research participants. MS, multiple sclerosis; NMOSD, neuromyelitis optica spectrum disorder; AD, Alzheimer’s dementia; N, number.

A total of 2,515 MS and 2,466 NMOSD patients from January 1, 2010 to December 31, 2019 were identified. The exclusion criteria were the following: (1) not registered to RID (MS, *N* = 9; NMOSD, *N* = 3); (2) died before the index date (MS, *N* = 1; NMOSD, *N* = 0); (3) previous history of dementia prior to MS or NMOSD diagnosis (MS, *N* = 129; NMOSD, *N* = 36) or within 1 year from index date of MS or NMOSD diagnosis (MS, *N* = 139; NMOSD, *N* = 76); (4) unavailable data for matching variables (MS, *N* = 8; NMOSD, *N* = 3); and (5) <40 years of age (MS, *N* = 882; NMOSD, *N* = 888). In total, 1,347 MS and 1,460 NMOSD patients were included (Figure 1).

In previous studies, dementia was shown to be associated with diabetes (Cheng et al., 2012), hypertension (Iadecola, 2014), and dyslipidemia (Fillit et al., 2008; Reitz, 2013). Therefore, MS and NMOSD cases were matched to controls not diagnosed with dementia and alive in the index year based on age, sex, index year, and the presence of hypertension, diabetes mellitus, or dyslipidemia. Five matched controls were selected for each MS and NMOSD case from the KNHIS database. Comorbidities were defined as follows based on claims data prior to the index date: diabetes mellitus (≥1 claim per year under ICD-10 codes E10–14 and ≥1 claim per year for the prescription record of antidiabetic medication), hypertension (≥1 claim per year under ICD-10 codes I10-I11, or ≥1 claim per year for the prescription record of antihypertensive drugs), and dyslipidemia (≥1 claim per year under ICD-10 code E78 and ≥1 claim per year for the prescription of a lipid-lowering medication). Consequently, the analysis included a total of 2,807 (1,347 for MS and 1,460 for NMOSD) matched controls. This research was approved by the Institutional Review Board (IRB) of the Samsung Medical Center (IRB No. SMC-2020-12-108).

### Study outcome definition and follow-up

The primary endpoint was newly diagnosed AD, vascular dementia, and other dementia during the follow-up period. Dementia was defined based on ICD-10 codes as AD (≥2 outpatient visits with codes F00 and G30), vascular dementia (≥2 outpatient visits with code F01), or other dementia (≥2 outpatient visits with codes F02, F03, F10.7, G23.1, G31.0, G31.1, or G31.8), and prescription of antidementia medication such as acetylcholine esterase inhibitors (rivastigmine, galantamine, or donepezil) or an *N*-methyl-d-aspartate receptor antagonist (memantine) (Jeong et al., 2023). Any dementia included AD, vascular dementia, and other dementia. Other dementia included dementia in diseases other than Alzheimer’s disease or cerebrovascular disease, unspecified dementia, residual and late-onset psychotic disorder due to alcohol or psychoactive substances, progressive supranuclear ophthalmoplegia, circumscribed brain atrophy including frontotemporal dementia, pick disease, and progressive isolated aphasia, senile degeneration of brain, not elsewhere classified excluding Alzheimer’s disease, and other specified degenerative diseases of nervous system including Lewy body dementia. Mean duration of follow-up after index date was 4.4 ± 2.3 years for MS and 4.2 ± 2.3 years for NMOSD patients and 4.8 ± 2.3 and 4.4 ± 2.2 years for their matched controls, respectively.

### Statistical analysis

For continuous variables, descriptive statistics are presented as means and standard deviations (SDs), and for categorical variables, as numbers (percentages). Student’s *t*-tests were used to compare the two groups for continuous variables and the χ^2^ test or Fisher’s exact test for categorical variables. Cox proportional hazards regression was used to estimate the incidence of dementia associated with the prevalent MS or NMOSD using a crude and multivariable-adjusted model. Three models were constructed: unadjusted (Model 1), age, sex-adjusted (Model 2), and multivariate (adjusted for age, sex, household income, and presence of diabetes mellitus, hypertension, or dyslipidemia; Model 3). Adjusted hazard ratios (aHRs) and 95% confidence intervals (CIs) were calculated. The proportionality assumption was tested using Schoenfeld residuals and was satisfactory.

Stratified analyses based on sex, age, and the presence of diabetes, hypertension, or dyslipidemia were conducted. P for interaction was calculated. Forest plots with the aHRs and 95% CIs based on subgroups were established. SAS statistical package version 9.4 (SAS Institute Inc., Cary, NC, USA) was used for all statistical analyses and a *P*-value ≤ 0.05 was considered statistically significant.

## Results

At baseline, matching variables were well distributed between MS and NMOSD patients and healthy controls (Table 1). In the MS group, 41.9% of patients were male and 20.8% were >65 years of age. In addition, 43.9% had hypertension as a co-morbidity, followed by dyslipidemia (31.3%) and diabetes mellitus (16.9%). MS patients were more likely to be in the lower income group than the matched controls (37.7 vs. 23.2%).

**TABLE 1:** Baseline characteristics of the study population.

	MS (*N* = 1,347)	Control^†^ (*N* = 6,735)	*P*-value*	NMOSD (*N* = 1,460)	Control^†^ (*N* = 7,300)	*P*-value*
Sex, male (*N*, %)	565 (41.9)	2,825 (41.9)	1	491 (33.6)	2,455 (33.6)	1
Age (years)	56.2 ± 10.6	56.2 ± 10.6	1	54.6 ± 9.7	54.6 ± 9.7	1
40–64	1,067 (79.2)	5,335 (79.2)		1,222 (83.7)	6,110 (83.7)	
≥65	280 (20.8)	1,400 (20.8)		238 (16.3)	1,190 (16.3)	
**Comorbidities (*N*, %)**						
Diabetes, yes	227 (16.9)	1,135 (16.9)	1	305 (20.9)	1,525 (20.9)	1
Hypertension, yes	591 (43.9)	2,955 (43.9)	1	427 (29.3)	2,135 (29.3)	1
Dyslipidemia, yes	422 (31.3)	2,110 (31.3)	1	454 (31.1)	2,270 (31.1)	1
Income, low (*N*, %)	508 (37.7)	1,563 (23.2)	<0.0001	335 (23.0)	1,540 (21.1)	0.12

Data are presented as mean ± standard deviation for numerical variables and number (percentage) for categorical variables. N, number; MS, multiple sclerosis; NMOSD, neuromyelitis optica spectrum disorder. Low income represents wages for the bottom 20%.

*χ^2^ test or fisher exact test for categorical variables, as appropriate.

^†^Matched for age, sex, income, and presence of diabetes, hypertension, or dyslipidemia.

In the NMOSD group, 33.6% of participants were male and 16.3% were >65 years of age. In addition, 31.1% had dyslipidemia as a co-morbidity, followed by hypertension (29.3%) and diabetes mellitus (20.9%). A difference was not observed in the income level between NMOSD patients and matched controls.

### Risk of dementia in MS and NMOSD patients compared with matched controls

During follow-up, the incidence of any dementia in MS patients was 17.08 per 1,000 person-years (aHR = 2.34; 95% CI = 1.84–2.96) compared with 7.36 for the matched controls; the incidence of AD was 13.19 per 1,000 person-years (aHR = 2.23; 95% CI = 1.70–2.91) compared with 6.03 for the matched controls; the incidence of vascular dementia was 2.54 per 1,000 person-years (aHR = 3.75; 95% CI = 1.91–7.35) compared with 0.68 for the matched controls. In NMOSD patients, the incidence of any dementia was 9.04 per 1,000 person-years (aHR = 2.19; 95% CI = 1.61–3.0) compared with 4.56 for the matched controls; the incidence of AD was 6.25 per 1,000 person-years (aHR = 1.99; 95% CI = 1.38–2.88) compared with 3.55 for the matched controls; the incidence of vascular dementia was 1.64 per 1,000 person-years (aHR = 3.21; 95% CI = 1.47–7.02) compared with 0.53 for the matched controls (Table 2).

**TABLE 2:** Incidence rates of dementia in MS and NMOSD patients.

	*N*	Events	Person-years	Incidence rate*	aHR^†^ (95% CI)
**Any dementia**					
Control	6,735	238	32,339.00	7.36	1 (Ref.)
MS	1,347	101	5,911.85	17.08	2.34 (1.84–2.96)
**AD**					
Control	6,735	195	32,339.00	6.03	1 (Ref.)
MS	1,347	78	5,911.85	13.19	2.23 (1.70–2.91)
**Vascular dementia**					
Control	6,735	22	32,339.00	0.68	1 (Ref.)
MS	1,347	15	5911.85	2.54	3.75 (1.91–7.35)
**Any dementia**					
Control	7,300	145	31,812.73	4.56	1 (Ref.)
NMOSD	1,460	55	6,084.29	9.04	2.19 (1.61–3.00)
**AD**					
Control	7,300	113	31,812.73	3.55	1 (Ref.)
NMOSD	1,460	38	6,084.29	6.25	1.99 (1.38–2.88)
**Vascular dementia**					
Control	7,300	17	31,812.73	0.53	1 (Ref.)
NMOSD	1,460	10	6,084.29	1.64	3.21 (1.47–7.02)

N, number; MS, multiple sclerosis; NMOSD, neuromyelitis optica spectrum disorder; AD, Alzheimer’s disease; aHR, adjusted hazard ratio; CI, confidence interval.

*Incidence rates of 1,000 person-years.

^†^Adjusted for age, sex, income, and the presence of diabetes, hypertension, or dyslipidemia.

### Difference in the incidence of dementia between MS and NMOSD patients

Compared with MS patients, NMOSD patients were less likely to have any dementia (aHR = 0.67; 95% CI = 0.47–0.94) and AD (aHR = 0.62; 95% CI = 0.41–0.93; Table 3). The risk of vascular dementia was comparable between MS and NMOSD patients.

**TABLE 3:** Difference in the incidence of dementia between MS and NMOSD patients.

Type of dementia	Disease	N	Event	Person-years	Incidence rate*	aHR^†^ (95% CI)
Any dementia	MS	1,347	101	5,911.85	17.08	1 (Ref.)
	NMOSD	1,460	55	6,084.29	9.04	0.67 (0.47–0.94)
AD	MS	1,347	78	5,911.85	13.19	1 (Ref.)
	NMOSD	1,460	38	6,084.29	6.25	0.62 (0.41–0.93)
Vascular dementia	MS	1,347	15	5,911.85	2.54	1 (Ref.)
	NMOSD	1,460	10	6,084.29	1.64	0.73 (0.32–1.69)

N, number; MS, multiple sclerosis; NMOSD, neuromyelitis optica spectrum disorder; aHR, adjusted hazard ratio; AD, Alzheimer’s disease; CI, confidence interval.

*Incidence rates of 1,000 person-years.

^†^Matched for age, sex, income, and the presence of diabetes, hypertension, or dyslipidemia.

### Risk of dementia based on age, sex, and comorbidities

**FIGURE 2:**
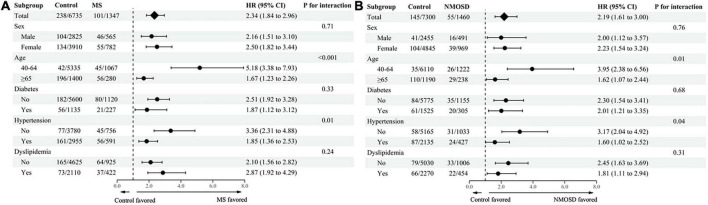
Subgroup analysis of any dementia in **(A)** MS and **(B)** NMOSD patients stratified based on age, sex, and comorbidities. MS, multiple sclerosis; NMOSD, neuromyelitis optica spectrum disorder; HR, hazard ratio; CI, confidence interval.

The higher relative risk of any dementia in MS or NMOSD patients tended to be more pronounced in patients 40–64 years of age than patients >65 years of age (aHR = 5.18; 95% CI = 3.38–7.93 vs. aHR = 1.67; 95% CI = 1.23–2.26, *P* for interaction <0.001 for MS; aHR = 3.95; 95% CI = 2.38–6.56 vs. aHR = 1.62; 95% CI = 1.07–2.44, *P* for interaction = 0.01 for NMOSD) and in patients who did not have hypertension compared with patients who had hypertension (aHR = 3.36; 95% CI = 2.31–4.88 vs. aHR = 1.85; 95% CI = 1.36–2.53, *P* for interaction <0.001 for MS; aHR = 3.17; 95% CI = 2.04–4.92 vs. aHR = 1.60; 95% CI = 1.02–2.52, *P* for interaction = 0.04 for NMOSD; Figure 2). Similarly, a higher risk of AD in MS or NMOSD patients tended to be more pronounced in patients 40–64 years of age than in patients >65 years of age (aHR = 6.17; 95% CI = 3.67–10.37 vs. aHR = 1.56; 95% CI = 1.12–2.19, *P* for interaction <0.001 for MS; aHR = 4.17; 95% CI = 2.17–8.00 vs. aHR = 1.48; 95% CI = 0.93–2.37, *P* for interaction = 0.01 for NMOSD) and in patients who did not have hypertension compared with patients who had hypertension (aHR = 3.31; 95% CI = 2.17–5.06 vs. aHR = 1.74; 95% CI = 1.22–2.47, *P* for interaction = 0.01 for MS; aHR = 3.49; 95% CI = 2.08–5.85 vs. aHR = 1.21; 95% CI = 0.69–2.13, *P* for interaction <0.001 for NMOSD; Supplementary Figure 1). However, statistically significant difference was not observed in relative risk of vascular dementia based on each subgroup in MS and NMOSD patients (Supplementary Figure 2).

## Discussion

In the present study, the risk of developing dementia was 2.34-fold higher in MS and 2.19-fold higher in NMOSD patients compared with matched controls. The increased risk of AD and vascular dementia in MS was 2.23- and 3.75-fold, respectively, and in NMOSD was 1.99- and 3.21-fold, respectively. MS patients had 1.49- and 1.61-fold higher risk of any dementia and AD than subjects with NMOSD, although the risk of vascular dementia did not significantly differ. To the best of our knowledge, this is the first population-based cohort study in which the risk of dementia in MS and NMOSD patients was examined.

Increased risk of AD was identified in MS patients (aHR = 2.23) compared with controls. AD was the most common type of dementia in the present study subjects (approximately 80% of all incident dementia cases). The coexistence of MS and AD has been reported in previous autopsy cases. In an 1994 autopsy study that included 45 MS cases, MS patients reportedly developed AD pathology at a similar incidence rate compared with age-matched controls (Dal Bianco et al., 2008). Later, 11 of 67 MS autopsy cases showed cortical lesions of amyloid plaques and neurofibrillary tangles, fulfilling the neuropathological diagnostic criteria for AD; however, only one of 28 control cases had confounding AD pathology (Frischer et al., 2009; Luczynski et al., 2019). However, knowledge regarding AD risk in MS patients is limited. In a recent large cohort study using 2007–2017 private claims data in the USA, MS increased the risk of AD and related dementias (aHR = 4.49 for patients 45–64 years of age and 1.26 for patients >65 years of age) (Mahmoudi et al., 2022), however, the risk of AD was not separately presented. In a UK cohort study, hospital admission for MS was associated with an elevated risk of future admission for any dementia including AD, vascular dementia, and unspecified dementia with a rate ratio of 1.97 (Wotton and Goldacre, 2017). In that study, the risk of hospitalization for AD (rate ratio = 0.95, 95% CI = 0.83–1.07) was not significantly increased; however, the study was limited to inpatients and the lack of information regarding comorbidities may have confounded the risk estimation (Wotton and Goldacre, 2017).

The association between MS and AD could be hypothesized from a shared pathophysiology. Blood-brain barrier (BBB) dysfunction and glymphatic system impairment affect the development of both MS and AD (Carotenuto et al., 2021; Scheltens et al., 2021). Microglia-mediated inflammation and mitochondrial dysfunction contribute to the deterioration of AD as well as neurodegeneration in MS (Xu et al., 2016). Cytoskeletal proteins, such as tau and neurofilaments, are thought to play critical roles in the pathology of AD and MS (Gutiérrez-Vargas et al., 2022). Apolipoprotein E (APOE) ε4 homozygosity, a strong risk factor for AD, was also suggested a potent predictor of cognitive decline in patients with early relapse-remitting MS (Engel et al., 2020). The reduced BBB clearance function of amyloid β (Aβ) associated with APOE ε4 burden may also apply to cognitive impairment in MS as in early AD (Uchida et al., 2022). Furthermore, MS patients may be more susceptible to the effects of Aβ deposition due to their preexisting brain damage, leading to a shorter preclinical stage of AD.

The risk of AD was also increased in patients with NMOSD (aHR = 1.99) compared with matched controls. In NMOSD, the target antigen of autoimmunity is the water channel aquaporin 4 (AQP4) densely expressed on the astrocytic foot processes at the blood-brain barrier (Jarius et al., 2020). In the animal model of AD, AQP4 deficiency increased amyloid load within the walls of brain vessels and surfaces of pia mater, which supports a facilitating role of AQP4 in the perivascular glymphatic drainage of Aβ (Xu et al., 2015). The depolarization of AQP4 in NMOSD may potentially contribute to the aggregation of Aβ by impairing its clearance and, thereby, have a detrimental role in the pathology of AD (Rasmussen et al., 2018). Antibody-mediated neuroinflammation and enhanced innate immunity in NMOSD may have detrimental effects on the development of AD (Jarius et al., 2020; Scheltens et al., 2021). However, to the best of our knowledge, comorbid NMOSD and AD has not been reported and their association should be further investigated.

In the present study, the risk of AD was relatively lower in NMOSD patients than in MS patients which may be due to distinctive mechanisms of neurodegeneration. Most patients with relapse-remitting MS experience a secondary progressive course with ongoing neurodegeneration (Kawachi and Lassmann, 2017). Conversely, in NMOSD, inflammatory relapse activity may be the main cause of neurodegeneration and secondary progression is very unlikely (Kawachi and Lassmann, 2017; Chen X. et al., 2021). In addition, patients with MS may have more severe neurodegeneration in WM lesions than subjects with NMOSD (Kato et al., 2022). However, whether disease-specific mechanisms contribute to AD pathology remains unknown.

For both MS and NMOSD subjects, younger patients (40–64 years of age) showed higher aHR for AD than older patients (≥65 years of age). It is known that advanced age is the strongest risk factor for AD and the majority of AD cases occur in individuals over the age of 65 (Scheltens et al., 2021). Therefore, our results may suggest that in younger patients, where the effect of age on AD risk is small, MS or NMOSD contributes to the increased risk of AD much more than in older patients. In addition, MS and NMOSD patients without hypertension had higher aHRs for AD than subjects with hypertension. In general, hypertension in midlife has been associated with an increased risk of AD in later life (Kivipelto et al., 2001; McGrath et al., 2017). Therefore, the significance of deleterious effects of MS or NMOSD on the development of AD could be less in individuals with hypertension, as shown in the present study.

Multiple sclerosis and NMOSD patients showed approximately 3–4-fold higher risk of vascular dementia than matched controls. The association between MS and vascular dementia was previously suggested in a UK study (Wotton and Goldacre, 2017); the hospitalization for MS was associated with an elevated risk of future admission for vascular dementia (rate ratio = 1.17, 95% CI = 1.04–1.32). Higher prevalence of vascular risk factors and stroke in MS and NMOSD may be associated with increased risk of vascular dementia (Marrie et al., 2015; Ajmera et al., 2018; Cho et al., 2022). However, in the absence of vascular comorbidities, vascular changes with periarteriolar inflammation and hemosiderin deposition were found even outside WM lesions in the MS brain (Geraldes et al., 2020). The accelerated small vessel disease may further contribute to the development of vascular dementia and the neurodegeneration of MS (Martinez Sosa and Smith, 2017). Previous research regarding NMOSD is limited and the role of MS and NMOSD in the pathogenesis of vascular dementia remains unclear.

Diagnosing dementia of other etiology is challenging in MS and NMOSD because the diseases can cause cortical atrophy and cognitive impairment (Benedict et al., 2020). The neuropsychological features of early cognitive impairment in MS or NMOSD patients are similar to vascular dementia, and are characterized by attention deficit and slowed information processing speed (Kalaria, 2018; Benedict et al., 2020). MRI observations at the first onset of MS are sometimes confused with findings of cerebral small vessel disease, the most common cause of vascular dementia (Kalaria, 2018). However, acute or subacute clinical onset and characteristic WM lesion patterns, such as Dawson’s finger and subcortical U fiber involvement, in MS can be distinguished from small vessel disease. The insidious onset and gradual worsening of episodic memory and mesial temporal lobe atrophy may indicate AD; however, this may also be found during the course of MS (Benedict et al., 2009). However, in Republic of Korea, most patients with MS or NMOSD have been regularly followed at tertiary hospitals and advanced images, such as amyloid positron emission tomography (PET), are widely available. Increased cortical signals on amyloid PET may indicate comorbid AD in MS patients with progressive cognitive impairment (Kolanko et al., 2020). Therefore, diagnosis of AD or vascular dementia in MS or NMOSD patients may be fairly accurate, although the diagnostic sensitivity of dementia may be higher in these patient populations due to surveillance bias. In addition, a MS specialist entering an AD code only to prescribe dementia drugs is very unlikely because evidence does not exist that such drugs are effective for cognitive symptoms of MS and NMOSD (Benedict et al., 2020). In the current clinical setting, careful review of neuroimaging and past medical history is recommended to differentiate MS-related cognitive impairment; however, the evaluation of dementia type in MS and NMOSD patients remains complicated and future studies using biomarkers, neuroimaging, and neuropathological findings are needed.

The present study has several limitations. First, because the data were claims-based, disease duration, disease activity, severity of vascular risk factors, smoking, alcohol consumption, physical activity, education level, and drug use data, which might also affect the risk of dementia in MS and NMOSD patients, were not available. Second, it is important to note that there may be a time lag between the onset of a disease and its inclusion in the National Health Insurance database with RID program benefits. That’s because clinicians should be cautious about enrolling patients in the RID program for substantial benefits of reduced payment. In addition, since anti-AQP4 antibody testing for medical use has been possible since 2015 in Republic of Korea, the patients who met the 2015 diagnostic criteria for NMOSD may have been included rather late in the disease process. This could partly explain the relatively short disease duration from MS and NMOSD to dementia diagnosis observed in this study. Third, there may be potential surveillance bias in this study. People with MS and NMOSD are more likely to be diagnosed with dementia than controls because they have more frequent access to healthcare. Finally, the accurate differentiation of AD or vascular dementia from cognitive decline caused by MS or NMOSD itself can be challenging in the database using the ICD-10 codes, although we used the same definition of AD and vascular dementia previously reported in Republic of Korea population study (Cho et al., 2022; Jeong et al., 2023). In the future, the coexistence or the risk of AD and vascular dementia should be investigated using molecular, imaging, and pathological tools in people with MS and NMOSD.

## Conclusion

The risk of dementia was higher in MS and NMOSD patients than in the general population and the dementia risk in MS patients was higher than in NMOSD subjects. Therefore, clinicians should be vigilant regarding cognitive impairment in patients with MS and NMOSD. Furthermore, it is important for clinicians to consider potential causes of dementia beyond MS/NMOSD itself, as preventive and therapeutic approaches vary depending on the underlying etiology. Further investigations should be performed to determine the pathophysiology of increased risk of AD or vascular dementia in MS and NMOSD patients.

## Data availability statement

The original contributions presented in this study are included in the article/Supplementary material, further inquiries can be directed to the corresponding authors.

## Ethics statement

The studies involving human participants were reviewed and approved by the Institutional Review Board (IRB) of the Samsung Medical Center (IRB No. SMC-2020-12-108). Written informed consent for participation was not required for this study in accordance with the national legislation and the institutional requirements.

## Author contributions

EC, SJ, DS, and J-HM: study conception and design, data analysis and interpretation, and manuscript drafting and revision. J-HJ and KH: data analysis and interpretation. YY and HK: data interpretation and critical review of manuscript. All authors contributed to the article and approved the submitted version.

## Footnotes

1
https://nhiss.nhis.or.kr/bd/ab/bdaba000eng.do
