# Supplementary material for: The risk of dementia in multiple sclerosis and neuromyelitis optica spectrum disorder
Source: Front Neurosci. 2023 Jun 15;17:1214652. doi: 10.3389/fnins.2023.1214652 (PMC10309000; doi:10.3389/fnins.2023.1214652)
Supplement: Supplementary file 1 [file Data_Sheet_1.docx]

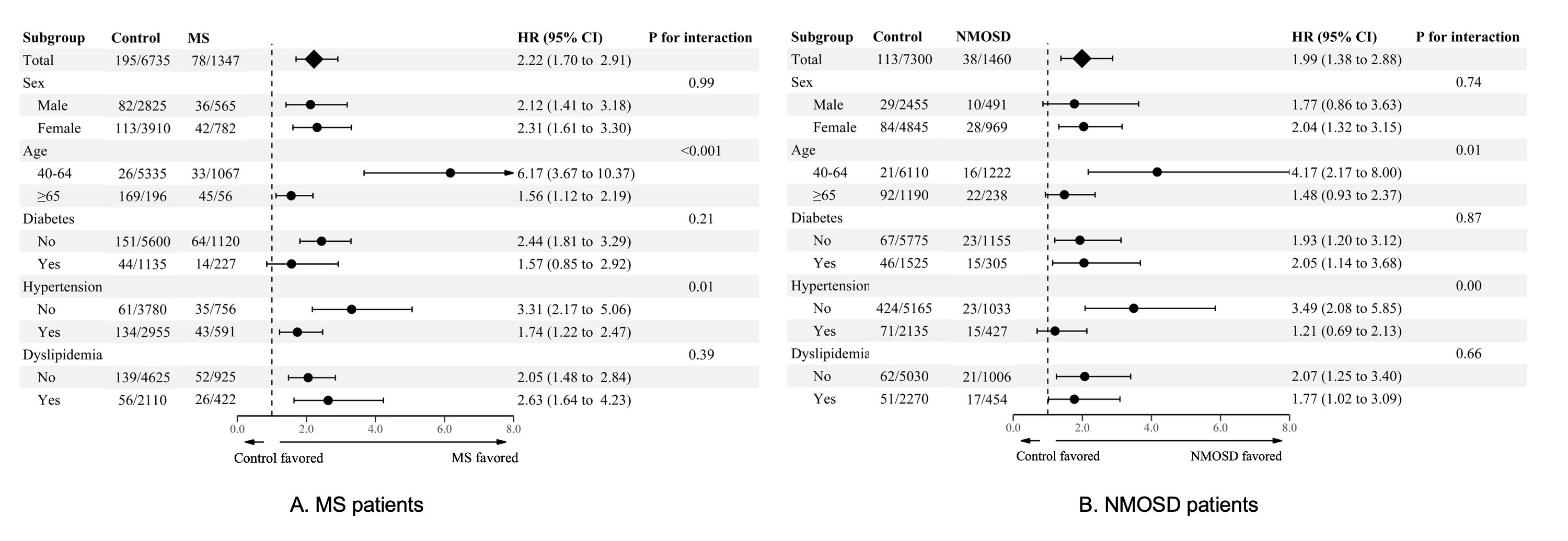


**Supplementary Figure 1**. Subgroup analysis of Alzheimer’s disease in MS and NMOSD patients stratified based on age, sex, and comorbidities

MS, multiple sclerosis; NMOSD, neuromyelitis optica spectrum disorder; HR, hazard ratio; CI, confidence interval


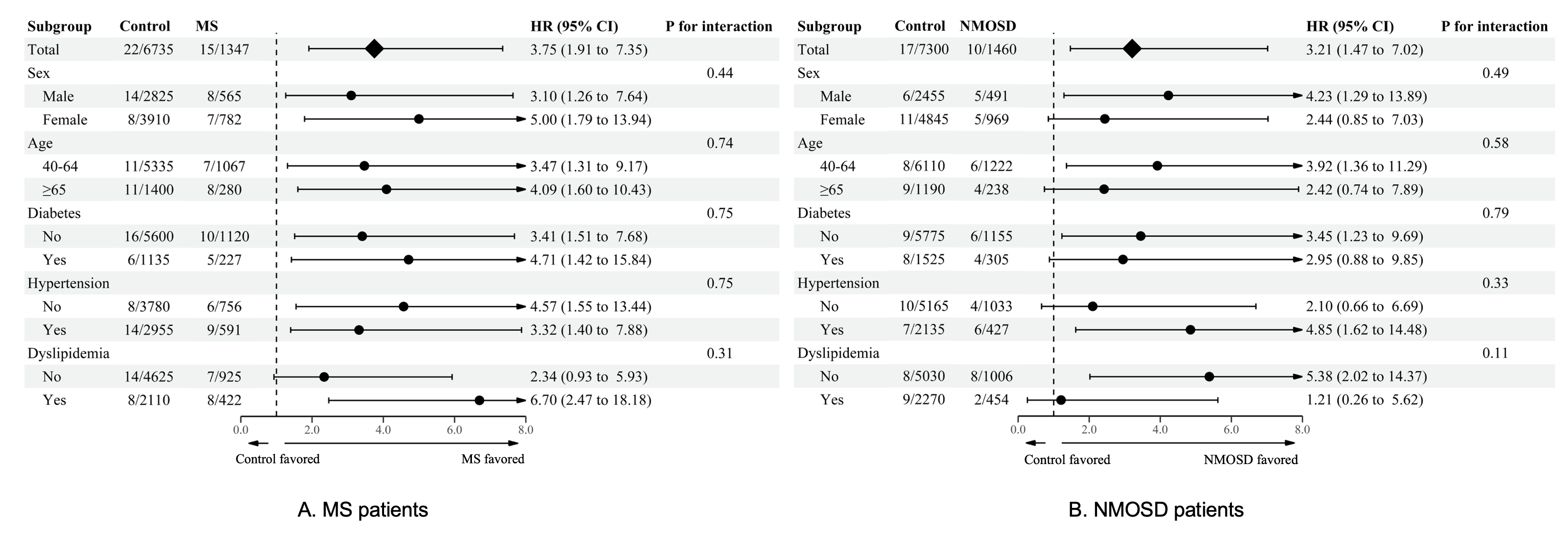


**Supplementary Figure 2**. Subgroup analysis of vascular dementia in MS and NMOSD patients stratified based on age, sex, and comorbidities

MS, multiple sclerosis; NMOSD, neuromyelitis optica spectrum disorder; HR, hazard ratio; CI, confidence interval
